# Biomechanical mechanism and clinical management progress of surgical wound tension

**DOI:** 10.3389/fsurg.2025.1674382

**Published:** 2025-09-22

**Authors:** Feiyu Gong, Bingjie Wan, Ping Qi, Zairong Wei

**Affiliations:** ^1^ Department of Burn and Plastic Surgery, Affiliated Hospital of Zunyi Medical University, Zunyi, Guizhou, China; ^2^ Emergency Intensive Care Unit, Affiliated Hospital of Zunyi Medical University, Zunyi, Guizhou, China; ^3^ The 2011 Collaborative Innovation Center of Tissue Damage Repair and Regeneration Medicine, Affiliated Hospital of Zunyi Medical University, Zunyi, Guizhou, China; ^4^ Guizhou Provincial Collaborative Innovation Center for Tissue Injury Repair and Regenerative Medicine, Affiliated Hospital of Zunyi Medical University, 2011, Zunyi, Guizhou, China

**Keywords:** surgical wound tension, wound healing, biomechanics, quantitative evaluation, clinical management

## Abstract

Surgical wound tension, a core biomechanical factor in tissue repair, is clinically important because high tension can cause microcirculatory disturbances, leading to inhibition of cell migration and collagen deposition, and increasing complications such as wound dehiscence and incisional hernia. Therefore, the concept of “active tension reduction” has been emphasized, including preoperative optimization of biomechanical distribution, intraoperative layered combined subcutaneous tension-reducing suturing, and postoperative dynamic management. However, the difficulty in standardizing wound tension quantification presents clinical challenges. In summary, this study integrates the biomechanical mechanisms of surgical wound tension with clinical practice to explore a systematic strategy from tension assessment to novel intervention techniques.

## Introduction

The regulation of wound tension can be traced back to 3,000 BC (1). With the widespread use of aseptic operation and anesthesia, this subjective judgment began to shift to the study of the biomechanical mechanism of tension (2). The advent of buried vertical mattress suture 100 years ago promoted the development of subcutaneous suture by redistributing tension to promote wound healing (3). The emergence of tensiometers can quantify the tension of the wound (4). With the optimization of suturing technology (5), the development of reverse traction devices (6) and new materials (7, 8), the understanding of tension has shifted from subjective judgment to the study of biomechanical mechanisms, providing strong support for the safety and aesthetics of wound healing.

Surgical wound tension plays a key role in the process of wound healing and scar formation and is a key factor affecting the quality of healing. High tension can cause separation of wound edges, reduce local blood supply, stimulate excessive proliferation of fibroblasts and disrupt collagen metabolism. This can lead to chronic inflammation, fibrosis and scar widening. In severe cases, it can induce hypertrophic scars or scar contracture deformities, affecting the patient's appearance and function (9). Mechanical tension has differential effects in the inflammatory, proliferative, and tissue remodeling phases of wound healing. This differential effect can induce biological responses such as chronic inflammation, fibrosis, angiogenesis, and extracellular matrix remodeling through complex signal transduction and feedback mechanisms, affecting wound healing and scar formation (10).

During the inflammatory phase, high tension can activate the integrin-focal adhesion kinase pathway, promote the release of proinflammatory factors (TNF-α, IL-1β, IL-6), and induce the activation of the proinflammatory signaling pathway (NF-κB), thereby prolonging the inflammatory phase and aggravating the local inflammatory microenvironment (11). In addition, the high-tension state of the wound can regulate the polarization of macrophages to M1, resulting in the inability to transition from the inflammatory phase to the proliferation phase, further aggravating the inflammatory response (12, 13). Excessive tension leads to tissue hypoxia, which in turn activates hypoxia-inducible factor, promotes the accumulation of reactive oxygen species（ROS）, aggravates oxidative stress and cell damage, and further prolongs the inflammatory phase (14, 15).

During the proliferation phase, high tension inhibits fibroblast proliferation, causing cell morphology changes and differentiation into myofibroblasts, leading to excessive deposition and disordered arrangement of type I collagen in the extracellular matrix (ECM). This excessive presence and abnormal deposition of myofibroblasts increases scar formation (16). At the same time, high tension can cause disordered collagen fiber arrangement, and a large amount of disordered collagen deposition leads to scar hyperplasia, resulting in scar hyperplasia (hypertrophic scars, keloids, etc.) (7). Excessive wound tension leads to insufficient local tissue perfusion, affecting the formation of new blood vessels, the insufficient growth of granulation tissue, and loose wound closure, increasing the risk of wound dehiscence and infection (17).

During the tissue remodeling stage, tension has a particularly significant effect on scar formation. Continuous tension can induce the secretion of profibrotic factors such as TGF-β, leading to collagen arrangement and scar formation (18, 19). In addition, the imbalance of ECM remodeling caused by high tension can also lead to complications such as secondary injury or chronic ulcers (20).

## Causes of surgical wound tension and its biomechanical mechanism for healing

**Figure 1:**
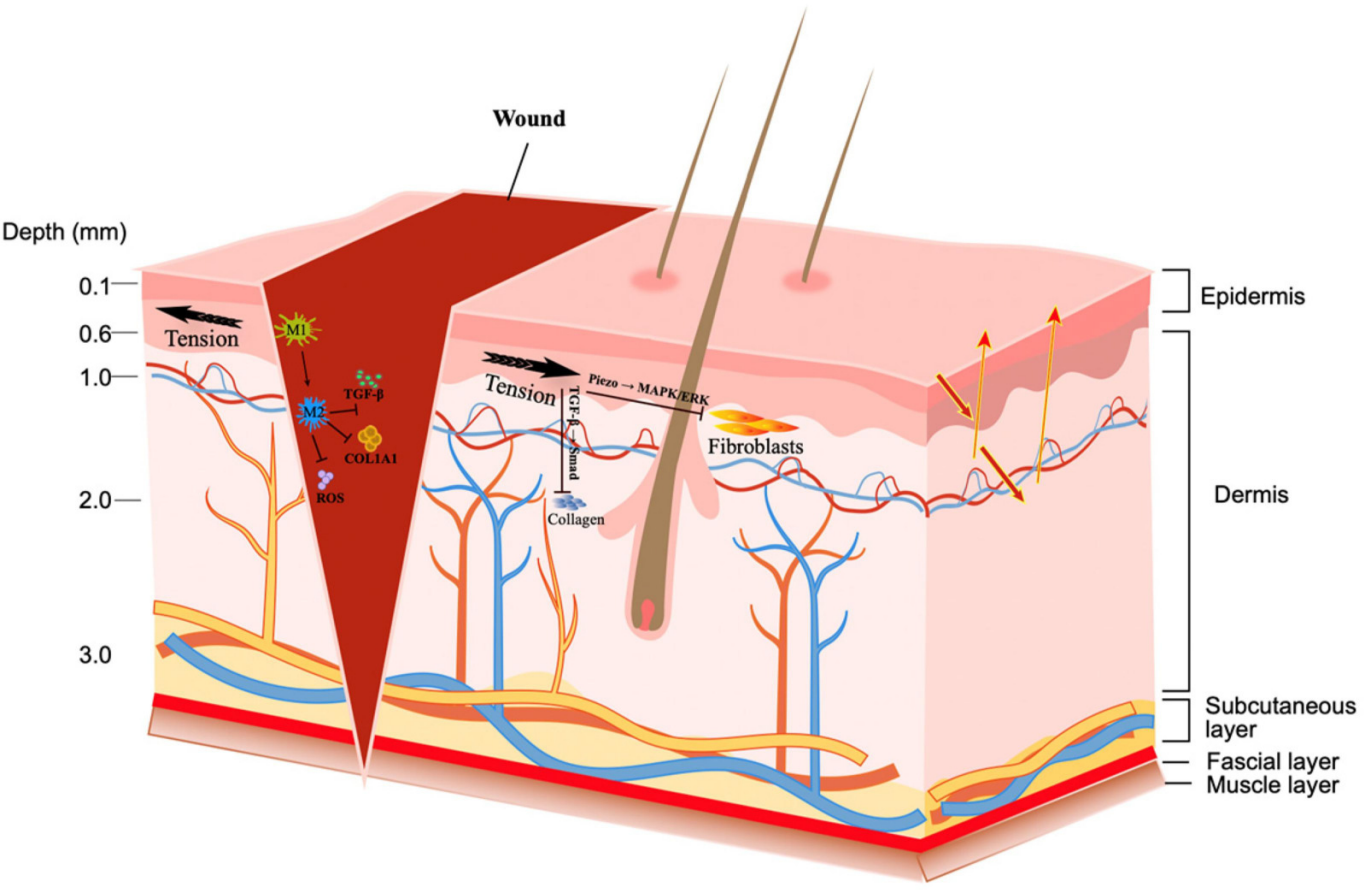
Surgical wound tension and its biomechanical mechanism for healing.

The tension on surgical wounds is the key to postoperative healing. Excessive tension can easily induce wound dehiscence, delayed healing, non-healing of wounds, abnormal proliferation of scar tissue, and infection (21). The formation of tension is caused by multiple factors such as the inherent properties of tissue structure, the specificity of anatomical regions, surgical operation methods, and postoperative care (22, 23) (Figure 1).

### Analysis of the causes of surgical wound tension

Tissue characteristics (such as collagen fibers, tissue tension, etc.) and anatomical structures (such as muscle fascia, ECM, etc.) can be considered the basis for tension formation. When the incision deviates from the axis of natural skin relaxation, the increased tension may cause the collagen structure in the dermis to be torn, and the retraction drive to increase dramatically (24). Especially in the facial and joint areas, the skin is thin and elastic. Once it moves, it will stimulate dynamic traction, making it difficult for the wound to heal (25). Large-area skin tears or deep structure damage (such as fascia) require strong mechanical tension to be forcibly resisted during suturing. Due to the lack of soft tissue buffer, stress concentration is more likely to occur under the action of mechanical tension in bone protrusions such as the heel and sacrum, resulting in prolonged wound healing (26). Similarly, rough sutures and lack of layered fixation will cause tension to be concentrated on the epidermis while the fascia will not bear the force; sutures that are too thin are easy to tear, and those that are too thick will cause strong inflammation. Postoperative management is a “continuous challenge” that affects high wound tension. Early or intense postoperative activities will increase the local mechanical tension of the wound, leading to wound dehiscence or scar widening (27). The failure to use braces to fix joints after surgery, the failure to use negative pressure devices properly after high-tension wound sutures, and the continued swelling of tissues caused by systemic factors of the patient (28) are factors that continuously affect wound healing and cannot be ignored.

### Effects of surgical wound tension on cells and tissues

The influence of tension mechanism on surgical wounds involves complex regulation at multiple levels, including the dynamic balance of cell behavior, molecular signaling pathways, and inflammatory response. When the tension is greater, the proliferation of fibroblasts can be inhibited. In addition, tension activates the Piezo1/2 channel (Piezo1/2, as mechanosensitive cation channels, are overexpressed in keloid myofibroblasts), which can inhibit the active migration of fibroblasts through the MAPK/ERK pathway (29, 30). The traction force of the wound surface is the power source for wound closure, but excessive tension induces abnormal matrix deposition and forms fibrosis (31, 32). Tension activates TGF-β1 through integrin β1, which in turn initiates the phosphorylation reaction of Smad2/3, increases the expression of collagens such as COL1A1 and COL3A1, and causes matrix accumulation (33–35). High tension also activates NOX2, increases ROS levels, and induces the release of NLRP3 and IL-1β, forming a vicious cycle of “tension-oxidation-inflammation” (36). In the early stage of inflammation, mechanical tension can induce macrophages to polarize toward the M1 type, and promote the release of a large number of inflammatory factors by activating the TLR4/MyD88/NF-κB axis (37, 38). At the same time, tension also inhibits the JAK2/STAT3 and TRAF6/NF-κB pathways, hindering the process of inflammation relief (39). In addition, high tension induces the expression of chemokines such as CXCL8, driving neutrophils to gather in the injured area, resulting in increased tissue inflammatory response (40, 41).

### The dynamic characteristics of tension distribution show diverse characteristics

Wound tension is in dynamic balance with the anatomical structure of the skin. The skin is composed of the epidermis, dermis, and subcutaneous tissue. The arrangement direction of collagen fibers in the dermis (i.e., Langer's lines) determines the distribution of local tension (42). The thickness of the fat layer also affects stress distribution and can buffer deep tension under vertical load. The fascia layer and muscle activity also participate in tension regulation. The fascia connects different tissues and maintains mechanical balance (43). Stress concentration at the wound edge will put fibroblasts in a hypertonic state, delay healing and aggravate fibrosis, and even affect tissue structure and function (44). Stress concentration can change the local mechanical microenvironment, disrupt cell migration, and ECM reconstruction (45). Increasing the strength of suture density can increase the tensile strength of the wound, especially in short incisions such as 3 mm, where the enhancement effect is more obvious (46). However, too dense sutures can also increase local stiffness and may also lead to poor tissue blood supply, affecting the repair effect (47). Suture density should be finely adjusted based on wound location, biomechanical requirements, and tissue characteristics. Future research needs to further reveal the three-dimensional structural relationship between suture density and tension distribution, and explore its stability and adaptability in a dynamic healing environment (48).

The figure shows the epidermis, dermis, subcutaneous tissue, fascia, and muscle layers. Once a wound is formed, the black arrow indicates the direction of tension. Reducing wound hypertension can modulate macrophage polarization toward M2, inhibiting the expression of TGF-β1, COL1A1, and ROS. Tension activates Piezo1/2 pathways, inhibiting active fibroblast migration through the MAPK/ERK pathway. Tension activates TGF-β1 via integrin β1, in turn initiating Smad2/3 phosphorylation, leading to collagen matrix accumulation.

### Method for quantitative assessment of surgical wound tension

Traditional tension judgment relies on the doctor's personal subjective experience and lacks systematic and objective quantitative evaluation. Wound size was once mistakenly believed to be positively correlated with tension size (49, 50). Quantification of tension is expected to be more objective and reliable for tension measurement and management. Surgical tension assessment is shifting from traditional mechanical methods to intelligent detection, and more technologies are being introduced into the clinic. In the future, dynamic tension monitoring is expected to be achieved by combining intelligent sensing technology (51). Currently, methods for quantitative assessment of surgical wound tension include wound tensiometers, finite element analysis, Fourier transform combined with topological imaging, and elastic wave analysis (Table 1).

**Table 1:** Method for quantitative assessment of surgical wound tension.

Method	Advantages	Disadvantages	References
Wound tensiometer	Measuring wound tension During surgery	Interference with wound microenvironment and biocompatibility	(7, 52, 53)
FEA	Comparison of stress distribution of different suture methods	Model generalization ability is limited to individual differences	(54–56)
FTCWTI	Quantifying skin tension and direction to assist in incision design	Complex wounds have poor adaptability	(57, 58)
EWA	Quantifying tissue tension	Unable to distinguish between deep and surface tension	(59–61)

Wound tensiometers can directly measure wound tension and monitor it in real time during surgery. By analyzing the mechanical threshold of ischemic injury and the critical point of hypertrophic scar formation observed in clinical practice (7, 52), the threshold of wound closure can be objectively quantified (for example, a threshold between 5.4 and 6.0 N is considered “safe”). However, it relies on contact measurement, which may interfere with the wound microenvironment, and the biocompatibility of long-term implantation remains a difficult issue (53).

The personalized modeling feature of finite element analysis (FEA) can be used to compare the stress distribution of different suture methods. such as Ding screw tension band is better than the traditional Kirschner wire method (54). FEA can also quantify aortic aneurysm wall stress (55) and oral graft design tissue stress (56), providing a basis for optimizing surgical strategies. However, the generalization ability of the model is limited to individual differences and requires more clinical data support.

Fourier transform combined with topological images（FTCWTI）can quantify skin tension and direction, assisting in incision design (57). Its multimodal imaging can quantify wound morphology, but it is less adaptable to complex wounds (such as deep ulcers) (58). Elastic wave analysis (EWA) quantifies tissue tension by measuring surface wave velocity without destroying the wound structure (59). When predicting clinically relevant tension values, the accuracy rate can reach over 85% (60). However, due to the influence of skin moisture and thickness, it cannot distinguish between deep and surface tension, and other technologies need to be combined (61).

In addition, Confocal microscopy can be used to evaluate living skin, but its applicability to chronic wounds still needs to be verified (62, 63). The distance between cells is regulated by the mechanical properties of the cells and the geometry of the wound, which indirectly reflects the tension state (64, 65). Gold nano-DNA probes can image intercellular tension and help quantify microscale mechanics (66). Objective quantitative assessment of tension will help scientifically evaluate the difficulty of wound healing and achieve personalized treatment.

### Clinical management strategies for surgical wounds

Clinical management of surgical wounds has shifted from traditional experience to precision medicine. The optimization and improvement of traditional technologies and the innovation of new intervention technologies have jointly opened up new wound healing goals (Table 2).

**Table 2:** Clinical management strategies for surgical wounds.

Methods	Research content	Application	References
Suture technique	Heart-shaped tension-reducing suture	Multicenter retrospective study of scars	(68)
	Zunyi suture	Application research in flap donor site suture	(69)
Tension reducing	RT-SSD	Primary closure of large skin defects	(71)
Device	NPWT	Tension wounds with high risk of infection	(72)
New technologies	FMTB	Promote cell migration and tissue regeneration	(81–83)
	Hydrogel	Formation of "cell bridge" drives osteogenic differentiation	(91)

Optimization and improvement of traditional technologies. Traditional technologies are centered on disinfection, debridement, and suturing. The exploration of super-tension-reducing suture methods plays a key role in long-term tension maintenance, scar minimization and functional recovery (67). Traditional methods (such as continuous suture and interrupted suture) have limited effects on long-term tension maintenance and scar control. New suturing techniques (such as heart-shaped tension-reducing suture and Zunyi suture) disperse stress more evenly than traditional techniques and reduce stress concentration (68, 69). Improvements in suture materials and structures can shorten operation time and improve tension resistance (70).

Traditional tension-reducing devices can provide a variety of solutions for high-tension wound healing, but they need to be selected according to wound type, location, and individual differences of patients. The reverse-traction skin-stretching device (RT-SSD) applies reverse tension to both sides of the wound by rotating the traction device, stretching collagen fibers, breaking elastin, inhibiting fibrosis, and thus reducing skin tension, especially for primary closure of large skin defects (71). Negative pressure wound therapy (NPWT) reduces edema, reduces incision line tension, enhances granulation tissue formation, reduces the risk of wound dehiscence and infection, and is suitable for tension wounds with high infection risk (72). Tension-reducing patches reduce wound edge tension by adhesion and prevent scar hyperplasia. After the use of negative pressure or stretching devices, tension-reducing patches can further maintain a low tension state.

New intervention technologies continue to promote innovation in wound management, especially showing potential in chronic wounds. Compared with traditional technologies, these technologies can achieve dynamic balance in the mechanical microenvironment, promote scarless healing, precisely fill complex wounds, control infection, and provide real-time monitoring capabilities (73–76).

Mechanical shielding hydrogels reduce external force interference through physical barriers, reduce tension and inflammation, and reduce scar formation (77, 78). Force-Modulating Tissue Bridges (FMTB) can serve as a hub for mechanical-biological coupling, and their application can be applied from basic mechanisms to clinical prognosis (79, 80). By combining the bioactivity and mechanical stability of hydrogels, they can simulate the natural microenvironment and promote cell migration and tissue regeneration (81–83). Bioactive hydrogel dressings have anti-infection, immunomodulatory, and angiogenic functions (84–86). Combining topological structures with drugs (such as lovastatin) can synergistically inhibit mechanical transduction and reduce fibrosis (87). 3D printed hydrogels can customize their structures and accurately load drugs/cells to adapt to complex wounds (88). Smart hydrogels can sense pH or enzyme changes, dynamically release drugs, and regulate tension distribution (89). Elastin hydrogels have shown reduced neutrophil recruitment, increased M2 macrophages, and increased numbers of newly formed hair follicles in a deep second-degree burn model (90). Studies have found that curvature fibers in hydrogels can promote the formation of “cell bridges” by enhancing cytoskeletal tension, bridging myosin-II in cells to generate directional forces and drive osteogenic differentiation (91). This mechanism can be extended to wound repair, guiding cell migration and tissue regeneration through mechanics.

## Challenges and future directions

At present, there is a lack of objective standards for assessing the tension of surgical wounds. The tension of different parts and ages varies greatly. Clinical practice relies more on subjective assessment or indirect indicators (such as fascial pressure, etc.) (92). Although FMTB and NPWT have certain potential, they are limited by high costs and a lack of long-term data. The suture anchoring method can induce scar models in mice (93), and the Retroflex model can simulate human pathological scars (94), which improves research efficiency. However, animal skin differs significantly from human skin in stiffness, thickness, and collagen content (95). Existing animal models cannot fully simulate the immune response and healing process of human skin (96). Although the diabetes model is used to study delayed healing, it is still difficult to fully simulate chronic ulcers (97).

Currently, clinical evidence is mostly retrospective or small-sample studies, and there is a lack of high-quality randomized controlled trials (RCTs) (98). Existing RCTs have a significant risk of publication bias, and comparative studies on wound closure effectiveness are mostly based on low-quality evidence (such as single-center quasi-RCTs) (99). Although new designs such as stepped-wedge RCTs (SW-RCTs), registry-based RCTs (RB-RCTs), and trials-within-cohorts (TwiCs) have been proposed and have certain advantages, they are still in the exploratory stage (100). The decision on which design is most suitable for each specific setting should be made on a case-by-case basis and expanded using the unified standard of reporting of trials.

Future development directions may be through multimodal evaluation, personalized treatment and interdisciplinary integration. The new tensiometer combines 3D and biomarker analysis to dynamically monitor the mechanical environment of the wound (101). Skin stiffness and viscoelasticity vary with individual differences. Increased stiffness or decreased viscoelasticity indicates scar formation or delayed healing (102). Further address the limitations of animal models by improving animal models, developing humanized technologies, and targeting mechanistic regulation strategies to promote clinical translation. Interdisciplinary integration is becoming a trend. In the future, we should focus on intelligent responsive materials, mechanical-biological coupling optimization, and the construction of a preclinical standardized evaluation system (103).

## Conclusion

The precise management of surgical wound tension must be based on biomechanical mechanisms. Tension is a key factor affecting healing quality and scar formation. Further research is needed to elucidate the cellular signaling networks of different tissue types (such as skin, fascia, and tendon) under dynamic tension. Through the development of quantitative tools (such as wound tensiometers, Fourier transform, FEA) and new technologies (such as hydrogels, FMTB), as well as suture technology optimization and individualized solutions guided by biomechanical models, we can ultimately achieve a paradigm shift from “passive closure” to “active regulation”, improve healing quality and reduce the risk of complications. Suturing techniques should be optimized and guided by biomechanical models to establish a personalized “patient-wound-procedure” approach. The future of surgical wound tension management lies in achieving precision, intelligence, and personalization, requiring multidisciplinary collaboration to achieve better patient outcomes.
